# Mini nutritional assessment short-form as a risk factor for mortality in patients with respiratory disease undergoing urgent hospitalization

**DOI:** 10.1186/s12877-025-05767-2

**Published:** 2025-02-19

**Authors:** Mayuko Ishiwari, Yuta Kono, Yuki Togashi, Kenichi Kobayashi, Ryota Kikuchi, Mariko Kogami, Ami Suekawa, Yasushi Miyazawa, Shinji Abe

**Affiliations:** 1 Department of Respiratory Medicine, Tokyo Medical University Hospital, 6-7-1 Nishi-Shinjuku, Shinjuku-ku, Tokyo, 160-0023 Japan; 2 Department of Nutrition Management, Tokyo Medical University Hospital, Tokyo, Japan

**Keywords:** Mini Nutritional Assessment Short-Form, MNA-SF, Mortality risk, Respiratory diseases

## Abstract

**Background:**

Studies of nutritional status in geriatric patients with respiratory diseases are limited. The aim of this study was to investigate the mortality risk of older patients undergoing urgent hospitalization for various respiratory diseases.

**Methods:**

This was a retrospective study of patients aged ≥ 65 years with respiratory diseases who were urgently hospitalized between April 2022 and November 2024. The Mini Nutritional Assessment Short-Form (MNA-SF) was evaluated at the time of urgent admission, and the malnutrition risk was defined by the MNA-SF score < 11. Comorbidities and frailty were assessed using the Charlson comorbidity index (CCI) and Clinical frailty scale (CFS), respectively. Biomarkers of inflammation and acute respiratory failure such as neutrophil-to-lymphocyte ratio (NLR), Glasgow Prognostic Score (GPS), Respiratory rate-oxygenation (ROX) index, and the pulse oximetric saturation (SpO_2_)/fraction of inspired oxygen (FiO_2_) [S/F] ratio were calculated and analyzed as risk factors of in-hospital mortality.

**Results:**

A total of 168 consecutive patients were enrolled in the study with median age of 77 years (interquartile range [IQR]: 72–84). Thirty-nine patients (23.2%) died during hospitalization, and the median time to death was 17 days (IQR: 10–25). Univariate analysis demonstrated that older age (> 77 years), low S/F ratio (< 315), low ROX (< 8.3), high NLR (> 6), high CFS (> 5), and low MNA-SF (< 11) were associated with in-hospital mortality, multivariate analysis revealed that low MNA-SF was an independent predictor.

**Conclusions:**

The MNA-SF is a risk factor for mortality in older patients undergoing urgent hospitalization due to various respiratory diseases.

## Background

The world’s population is aging, with 9.3% (730 million people) currently older than 65 years [1]. Japan’s population is aging rapidly, with 28.9% of the population being aged 65 and older in 2021 [2]. Concurrently, medical costs for the over 65 age group continue to increase due to hospitalization costs [3]. In older patients, nutritional status is often poor on admission, which may adversely affect clinical outcomes. Malnourished older adults have decreased immunity, increased susceptibility to infection [4], and decreased physical function [5], resulting in longer hospital stays [4] and frequent readmissions. Nutritional status has been reported to affect clinical outcomes in patients with chronic respiratory diseases, including chronic obstructive pulmonary disease (COPD) [6] and asthma [7].

The assessment of nutritional status holds particular significance for the early detection of malnutrition, identification of risk situations, and implementation of appropriate measures. Nutritional assessment methods specifically for older patients are becoming increasingly important, and the Mini Nutritional Assessment (MNA) is used in many countries and regions around the world [8]. The Mini Nutritional Assessment Short-Form (MNA-SF), which screens using the first six items of the MNA, is a simple and reportedly useful method of nutrition screening for hospitalized patients [9].

The objective of this study was to investigate the association between nutritional status determined using the MNA-SF and outcomes in patients over 65 years urgently admitted to the local respiratory center.

## Methods

We conducted a retrospective study including patients with pulmonary disease aged ≥ 65 years with dyspnea and a risk of respiratory failure who were urgently admitted to the Department of Respiratory Medicine at Tokyo Medical University Hospital from April 2022 to November 2024. This study was approved by the Institutional Review Board of Tokyo Medical University Hospital (Approval No. T2023-0093). Informed consent was waived, as the study involved a retrospective chart review with minimal risk to the patients. All data were anonymized before analysis.

### Collection of clinical data

Blood pressure, oxygen saturation, oxygen demand, and respiratory rate at admission were obtained from medical records. The following laboratory parameters or respiratory indexes measured at the time of admission were obtained from the medical records: albumin (Alb), white blood cell (WBC), absolute neutrophil count (ANC), absolute lymphocyte count (ALC), hemoglobin (Hb), C-reactive protein (CRP), and D dimer. Neutrophil-to-lymphocyte ratio (NLR), a systemic inflammatory index, was calculated as according to the following formula: NLR = ANC/ALC. Glasgow Prognostic Score (GPS) was classified into three groups according to blood CRP and Alb levels as reported previously [10]. The pulse oximetric saturation (SpO_2_)/fraction of inspired oxygen (FiO_2_) [S/F] ratio was used instead of the partial pressure of arterial oxygen (PaO_2_)/FiO_2_ (P/F) ratio to evaluate the degree of hypoxemia in patients with pneumonia, acute respiratory distress syndrome (ARDS), and acute lung injury [11]. The respiratory rate-oxygenation (ROX) index was defined as the ratio of S/F and respiratory rate (RR) and is conveniently used for bedside assessment [12]. Comorbidities and frailty were assessed using the Charlson comorbidity index (CCI) and Clinical frailty scale (CFS), respectively [13, 14].

### Nutritional assessment

A registered dietitian interviewed each patient at the time of their admission to the hospital. Nutritional status was assessed using the MNA-SF, a condensed form of the MNA designed specifically for screening older people [15]. In addition to incorporating body mass index (BMI), which can be replaced by calf circumference, it includes information on relevant variables in the last 3 months, such as reduced food intake, weight loss, mobility, psychological stress, occurrence of acute illness, or neuropsychological problems. The MNA-SF screening test consists of six questions, with scoring as follows: 11–14 points for normality, 8–10 points for risk of malnutrition, and 0–7 points for malnutrition. In our study, we analyzed the nutritional status variable dichotomized as follows: normal nutritional status (11–14 points) and risk of malnutrition (0–10 points) [16]. In the current study, height or weight could not be assessed in 16 patients, so calf circumference was measured as a surrogate for BMI as described previously [17].

### Statistical analysis

Data are described as number (percentage) or median (interquartile range [IQR]). Factors favoring survival after hospitalization were identified by both univariate and multivariate analysis using Cox proportional hazards models. Group comparisons were performed using the Mann-Whitney test for continuous variables and Fisher’s exact test for categorical variables. Probability values < 0.05 were considered statistically significant. All statistical analyses were performed using EZR (version 1.54) [18].

## Results

### Patients’ baseline characteristics

In total, 168 consecutive patients were included in this study. The clinical characteristics of the patients are shown in Table 1. The median age was 77 years (IQR: 72–84) and 113 patients (67.3%) were male. One hundred sixteen (69.0%) were ex-smokers. Respiratory diseases that led to urgent hospitalization included 86 cases of pneumonia (72 cases of bacterial pneumonia, 12 cases of COVID-19, 1 case of tuberculosis, 1 case of pulmonary aspergillosis), 34 cases of lung cancer, 30 cases of interstitial lung disease (ILD), 4 cases of COPD, 3 cases each of pneumothorax and asthma, 2 cases each of pulmonary malignant lymphoma, pulmonary embolism and hemoptysis due to bronchiectasis, 1 case each of pulmonary artery hypertension and allergy to antituberculosis drugs. Thirty-nine patients (23.2%) died during hospitalization, and the median time to death was 17 days (IQR: 10–25). Thirty patients died of respiratory failure, 5 of cancer progression, and 4 of multiple organ failure. The median values of S/F ratio and ROX index were 419 (IQR: 342–452) and 20.0 (IQR: 14.8–25.0), respectively. The median BMI was 21 kg/m² (IQR: 18–23). The median NLR was 7.3 (IQR: 4.3–13.1). The number of patients categorized into GPS 0, 1, and 2 was 16, 44, and 108, respectively. The median CCI was 6 (IQR: 5–9). The median CFS was 4 (IQR: 3–6). The median MNA score was 9 (IQR: 7–11) and 108 patients (64.3%) had MNA scores of less than 11. Malnutrition with MNA-SF score of 0–7 was present in 43 cases (25.6%) and severe dementia or depression was observed in 4 cases (2.4%).

**Table 1:** Clinical characteristics of the patients at admission (*n* = 168)

Variable	Value median (IQR)
**Demographic variables**	
Age, y	77 (72–84)
Gender	
Female	55 (32.7%)
Male	113 (67.3%)
Smoking	
ex-smoker	116 (69.0%)
never-smoker	52 (31.0%)
**Disease**	
Pneumonia	86
Lung Cancer	34
ILD	30
COPD	4
Pneumothorax	3
Asthma	3
Pulmonary malignant lymphoma	2
Pulmonary embolism	2
Hemoptysis due to bronchiectasis	2
Pulmonary artery hypertension	1
Allergy to antituberculosis drugs	1
**Outcome**	
death	39 (23.2%)
survival	129 (76.8%)
**Vital signs at admission**	
SpO_2_/FiO_2_ rate	419 (342–452)
Respiratory rate, /min	20 (18–24)
ROX index	20.0 (14.8–25.0)
SBP, mmHg	126 (109–143)
DBP, mmHg	76 (64–87)
BMI, kg/m² (*N* = 152)	21 (18–23)
**Laboratory variables**	
Hemoglobin, g/dL	11.8 (10.4–13.5)
WBC, /µL	9100 (7300–12800)
Neutrophil count, /µL	7117(5425–10884)
Lymphocyte count, /µL	1050 (673–1366)
NLR	7.3 (4.3–13.1)
CRP, mg/dL	6.5 (2.2–12.3)
Albumin, g/dL	3.2 (2.7–3.5)
GPS (0/1/2)	16 (9.5%)/44 (26.2%)/108 (64.3%)
D-dimer, µg/mL(*N* = 158)	2.7 (1.7–5.3)
**Charlson Comorbidity Index**	6 (5–9)
**Clinical Frailty Scale**	4 (3–6)
**MNA-SF score**	9 (7–11)
MNA SF nutritional status	
MNA-SF < 11	108 (64.3%)
MNA-SF ≧ 11	60 (35.7%)

*ILD* interstitial lung disease, *COPD* chronic obstructive pulmonary disease, *SpO*_*2*_ Pulse oximetric saturation, *FiO*_*2*_ Fraction of inspired oxygen, *ROX* respiratory rate-oxygenation, *SBP* systolic blood pressure, *DBP d*iastolic blood pressure, *BMI* Body mass index, *WBC* white blood cell, *NLR* Neutrophil-to-lymphocyte ratio, *CRP* C-reactive protein, *GPS* Glasgow Prognostic Score, *MNA-SF* Mini Nutritional Assessment Short-Form

Table 2 shows the bivariable comparison of patients who died in-hospital (*n* = 39) and those who recovered to discharge (*n* = 129). There were significant differences in age (*p* = 0.0218), causing disease (pneumonia *p* = 0.0435, ILD *p* = 0.0294), vital sigh at admission (S/F ratio *p* = 0.00015, RR *p* < 0.0001, ROX *p* < 0.0001), laboratory variables (WBC *p* = 0.0231, neutrophil count *p* = 0.0044, NLR *p* = 0.0162, Alb *p* = 0.0295, D-dimer *p* = 0.0294), CCI (*p* = 0.0406), CFS (*p* = 0.0006), and MNA-SF score (*p* < 0.0001).

**Table 2:** Bivariable comparison of patients who died in-hospital (death) with those who recovered to discharge (survival)

Variable	Death (*n* = 39)	Survival (*n* = 129)	*p* value
	Value median (IQR)		
**Demographic variables**			
Age, y	81 (75–87)	77 (72–82)	0.0218
Gender			0.174
Female	9	46	
Male	30	83	
Smoking			0.244
ex-smoker	30	86	
never-smoker	9	43	
Length of hospital stay	17 (10–25)	16 (11–18)	0.875
**Disease**			
Pneumonia	14	72	0.0435
Lung Cancer	12	22	0.0714
ILD	12	18	0.0294
COPD	0	4	0.574
Pneumothorax	0	3	0.564
Asthma	0	3	1
Pulmonary malignant lymphoma	1	1	0.411
Pulmonary embolism	0	2	1
Hemoptysis due to bronchiectasis	0	2	1
Pulmonary artery hypertension	0	1	1
Allergy to antituberculosis drugs	0	1	1
**Vital signs**			
SpO_2_/FiO_2_ rate	328 (212–428)	428 (375–466)	0.00015
Respiratory rate, /min	24 (20–29)	18 (18–24)	< 0.0001
ROX index	13 (7–19)	22 (17–25)	< 0.0001
SBP, mmHg	125 (105–136)	127 (112–144)	0.214
DBP, mmHg	80 (69–89)	74 (64–87)	0.21
BMI, kg/m² (*N* = 152)	19 (18–21)	21 (18–23)	0.148
**Laboratory variables**			
Hemoglobin, g/dL	11.8 (10.5–13.0)	11.9 (10.4–13.6)	0.393
WBC, /µL	10,500 (8350–14200)	8900 (7100–12200)	0.0231
Neutrophil count, /µL	8197 (6721–12165)	6638 (5090–10513)	0.0044
Lymphocyte count, /µL	1050 (602–1340)	1048 (720–1362)	0.41
NLR	8.6 (6.9–13.3)	6.5 (3.9–13.0)	0.0162
CRP, mg/dL	6.5 (2.2–12.3)	6.6 (2.3–12.3)	0.842
Albumin, g/dL	3.0 (2.5–3.3)	3.2 (2.8–3.5)	0.0295
GPS (0/1/2)	1/9/29	15/35/79	0.181
D-dimer,µg/mL (*N* = 158)	3.1 (2.4–6.5)	2.5 (1.5–4.7)	0.0294
**Charlson Comorbidity Index**	7 (6–10)	6 (5–8)	0.0406
**Clinical Frailty Scale**	5 (4–6)	4 (3–5)	0.0006
**MNA-SF score**	8 (5–9)	10 (8–12)	< 0.0001
MNA-SF nutritional status			< 0.0001
MNA-SF < 11	37	71	
MNA-SF ≧ 11	2	58	

*ILD* interstitial lung disease, *COPD* chronic obstructive pulmonary disease, *SpO*_*2*_ Pulse oximetric saturation, *FiO*_*2*_ Fraction of inspired oxygen, *ROX* respiratory rate-oxygenation, *SBP* systolic blood pressure, *DBP d*iastolic blood pressure, *BMI* Body mass index, *WBC* white blood cell, *NLR* Neutrophil-to-lymphocyte ratio, *CRP* C-reactive protein, *GPS* Glasgow Prognostic Score, *MNA-SF* Mini Nutritional Assessment Short-Form

### Outcomes

We determined whether the risk of malnutrition, as defined by the MNA-SF, was associated with a higher risk of mortality. Univariable analysis demonstrated that older age (> 77, hazard ratio [HR] = 0.443, 95% confidence interval [CI]: 0.211–0.930, *p* = 0.0314), low S/F ratio (< 315) (HR = 6.71, 95% CI: 2.96–15.2, *p* < 0.0001), low ROX index (< 8.3) (HR = 6.67, 95% CI: 2.58–17.2, *p* < 0.0001), high NLR (> 6.0) (HR = 0.326, 95% CI: 0.139–0.764, *p* = 0.0099), high CFS (> 5) (HR = 0.373, 95% CI: 0.179–0.776, *p* = 0.0008) and low MNA (< 11) (HR = 15.1, 95% CI: 3.49–65.4, *p* = 0.0003) were significantly associated with in-hospital mortality (Table 3). In the multivariate analysis, only low MNA score (HR = 14, 95% CI: 2.91–67.8, *p* = 0.001) had a significant effect on the prognosis of respiratory diseases in patients who were urgently hospitalized (Table 3).

**Table 3:** In-hospital mortality factors in the patients with urgent hospitalization

Variable	Hazard ratio	95%CI	*P* value	Hazard ratio	95%CI	*P* value
Univariate Cox analysis				Multivariate Cox analysis		
Older age (> 77 years)	0.443	0.211–0.930	**0.0314**	0.886	0.201–3.89	0.872
Gender (male)	0.541	0.237–1.240	0.146			
Smoking (ex-smoker)	1.67	0.727–3.82	0.228			
BMI (< 21)	1.97	0.909–4.29	0.0858			
SpO_2_/FiO_2_ rate (< 315)	6.71	2.96–15.2	**< 0.0001**	3.3	0.987-11	0.0526
ROX index (< 8.3)	6.67	2.58–17.2	**< 0.0001**	2.91	0.648–13.1	0.163
NLR (> 6.0)	0.326	0.139–0.764	**0.0099**	0.423	0.158–1.13	0.0856
Hemoglobin (< 11.9)	1.07	0.522–2.19	0.855			
D-dimer (> 2.8)	0.515	0.244–1.09	0.0814			
serum CRP (> 6.5)	0.995	0.486–2.04	0.99			
serum Alb (< 3.2)	2.15	0.985–4.67	0.0547			
GPS (< 2)	0.545	0.244–1.21	0.137			
Charlson Co-morbidity Index (> 6)	0.58	0.253–1.33	0.197			
Clinical Frailty Scale (> 5)	0.373	0.179–0.776	**0.0008**	0.607	0.248–1.49	0.275
MNA-SF score (< 11)	15.1	3.49–65.4	**0.0003**	14	2.91–67.8	**0.001**

*BMI* Body mass index, *SpO*_*2*_ Pulse oximetric saturation, *FiO*_*2*_ Fraction of inspired oxygen, *ROX* respiratory rate-oxygenation, *NLR* Neutrophil-to-lymphocyte ratio, *CRP* C-reactive protein, *Alb* albumin, *GPS* Glasgow Prognostic Score, *MNA-SF* Mini Nutritional Assessment Short-Form

## Discussion

This retrospective study addressed the importance of nutritional status in older patients with urgent hospitalization in various respiratory diseases. We demonstrated that risk of malnutrition, as evaluated by the MNA-SF, was significantly associated with in-hospital mortality in patients aged > 65 who were admitted urgently to the respiratory center.

Global surveillance shows that respiratory diseases such as pneumonia, lung cancer, COPD, and ILD are increasing and are leading causes of death in older adults [19]. Malnutrition is an important problem in older adults and has been shown to be a predictor of outcome in age-related respiratory diseases [20].

The MNA was developed to provide a single, rapid assessment of malnutrition in geriatric populations in hospitals, outpatient clinics, and nursing homes worldwide [21]. However, it is complex and too long for use as a screening tool in primary care or an emergency setting. The MNA-SF, using just the first six items of the MNA appetite or eating problems in the past 3 months, mobility impairment, acute illness/stress, dementia or depression and BMI is simple to administer and has been reported to be useful as a method of nutrition screening for hospitalized patients [9]. The MNA-SF has been recommended by the European Society for Clinical Nutrition and Metabolism (ESPEN) as a validated tool for the diagnosis of malnutrition and for predicting clinical outcomes [22]. The MNA-SF was able to predict not only cognitive and functional decline but also all-cause mortality in older men living in a veteran retirement community [23]. The MNA-SF is also significantly associated with long-term mortality and was an effective predictor of mortality in older adults in outpatient settings and nursing homes [24, 25]. In patients with chronic respiratory diseases, malnutrition or at risk of malnutrition, the MNA-SF has been reported to affect clinical courses or outcomes, mainly in patients with COPD and lung cancer [26–29]. However, no studies have used the MNA-SF to assess mortality risk factors in the acute phase of respiratory diseases. This is the first study to evaluate the MNA-SF in older patients undergoing urgent hospitalization for various respiratory diseases. In the present study, 62% of patients at the time of urgent admission were at risk of malnutrition, as evaluated by the MNA-SF. MNA-SF scores in patients who died during hospitalization were significantly lower than those in patients who recovered to discharge. The MNA-SF was an independent factor and more sensitive to in-hospital mortality than inflammation markers or respiratory failure indexes such as NLR, S/F ratio, and ROX, suggesting the usefulness of MNA-SF for estimating in-hospital mortality.

The superiority of the MNA-SF as a factor for in-hospital mortality may be explained by several studies. Gu et al. reported that a higher level of inflammation was observed in patients with poor nutritional status related to COVID-19 (Omicron) assessed by MNA-SF [30]. The MNA-SF score has been reported to be significantly associated with pulmonary function parameters including predicted forced expiratory volume in one second (%FEV1.0), predicted vital capacity (%VC), predicted residual volume (%RV), and predicted diffusing capacity of the lung for carbon monoxide (%DLco). Furthermore, it was correlated with muscle mass and strength in patients with COPD [26, 31]. Both pulmonary function and swallowing function were significantly associated with nutritional status determined by MNA-SF in older Japanese adults [32]. From these reports, MNA-SF may reflect systemic inflammation and physical activity such as pulmonary or swallowing function in the clinical setting. In the present study, 12 of 30 (40%) patients with ILD died during hospitalization. The proportion of in-hospital deaths tended to be higher in patients with ILD than in patients with other respiratory diseases. Lower BMI and body weight loss were associated with faster decline in forced vital capacity (FVC) and survival in patients with idiopathic pulmonary fibrosis (IPF) [33, 34]. Malnutrition may be caused by various conditions including chronic inflammation, oxidative stress, and reduced food intake due to muscle loss or inactivity, which could ultimately be associated with progression of IPF [34].

Several limitations of the present study should be considered. First, this was a single-center retrospective study with a small sample size. The small number of patients limited the power of multivariable analysis to evaluate in-hospital mortality factors. Second, the present study included older patients with various respiratory diseases. Nutritional status probably differed with the stage or severity of each respiratory disease. Additionally, the decision for urgent hospitalization was made by attending doctors, which introduces bias. Third, the present study assessed nutritional status using the MNA-SF only. Other nutritional evaluation tools and interventions will be needed to establish the usefulness of MNA-SF in respiratory diseases.

Despite these limitations, the present study demonstrated, for the first time, an association between nutritional status by MNA-SF and in-hospital mortality. Our findings provide useful information for managing respiratory diseases in patients undergoing urgent hospitalization using comprehensive geriatric evaluation.

## Conclusions

Malnutrition determined by the MNA-SF was independently associated with in-hospital mortality in older patients undergoing urgent hospitalization. The MNA-SF can be quickly utilized to evaluate nutritional status in older adults and estimate their prognosis in the clinical setting; however, larger prospective studies and active nutritional interventions will be needed.

## Data Availability

Data is provided within the manuscript.The datasets generated in this study are available from the corresponding author upon reasonable request.

## Declarations

### Rights and permissions

Open Access.

## Abbreviations

Alb: Albumin
ALC: Absolute lymphocyte count
ANC: Absolute neutrophil count
ARDS: Acute respiratory distress syndrome
BMI: Body mass index
CCI: Charlson comorbidity index
CFS: Clinical frailty scale
COPD: Chronic obstructive pulmonary disease
CRP: C-reactive protein
DBP: Diastolic blood pressure
FiO_2_: Fraction of inspired oxygen
FVC: Forced vital capacity
GPS: Glasgow Prognostic Score
Hb: Hemoglobin
ILD: Interstitial lung disease
IPF: Idiopathic pulmonary fibrosis
MNA: Mini Nutritional Assessment
MNA-SF: Mini Nutritional Assessment Short-Form
NLR: Neutrophil-to-lymphocyte ratio
PaO_2_: Partial pressure of arterial oxygen
P/F: PaO_2_/FiO_2_
ROX: Respiratory rate-oxygenation
RR: Respiratory rate
SBP: Systolic blood pressure
S/F: SpO_2_/FiO_2_
SpO_2_: Pulse oximetric saturation
WBC: White blood cell
%DLco: Predicted diffusing capacity of the lung for carbon monoxide
%FEV1.0: Predicted forced expiratory volume in one second
%RV: Predicted residual volume
%VC: Predicted vital capacity
